# Metabolic Profiling of *Buddleia indica* Leaves using LC/MS and Evidence of their Antioxidant and Hepatoprotective Activity Using Different In Vitro and In Vivo Experimental Models

**DOI:** 10.3390/antiox8090412

**Published:** 2019-09-18

**Authors:** Fadia S. Youssef, Mohamed L. Ashour, Hesham A. El-Beshbishy, Abdel Nasser B. Singab, Michael Wink

**Affiliations:** 1 Department of Pharmacognosy, Faculty of Pharmacy, Ain-Shams University, Cairo 11566, Egypt; fadiayoussef@pharma.asu.edu.eg (F.S.Y.); ashour@pharma.asu.edu.eg (M.L.A.); 2 Pharmacy Program, Batterjee Medical College, North Obhur, P.O. Box 6231, Jeddah 21442, Saudi Arabia; 3 Medical Laboratory Sciences Department, Fakeeh College for Medical Sciences, Jeddah 21461, Saudi Arabia; hesham_elbeshbishy@hotmail.com; 4 Biochemistry Department, Faculty of Pharmacy, Al-Azhar University, Cairo 11231, Egypt; 5 Institute of Pharmacy and Molecular Biotechnology, Heidelberg University, INF 364, D-69120 Heidelberg, Germany

**Keywords:** antioxidant activity, *Buddleia indica*, HepG2 cells, hepatoprotective activity, scrophulariaceae, molecular modeling

## Abstract

LC-ESI-MS (Liquid Chromatography coupled with Electrospray Ionization Mass Spectrometry profiling of a methanol extract from *Buddleia indica* (BIM) leaves revealed 12 main peaks in which verbascoside and buddlenoid B represent the major compounds. The antioxidant and hepatoprotective activities of BIM were investigated using different in vitro and in vivo experimental models. BIM exhibited substantial in vitro antioxidant properties in DPPH· and HepG2 assays. Regarding CCl_4_ (carbon tetrachloride) induced hepatotoxicity in a rat model, oxidative stress markers became significantly ameliorated after oral administration of BIM. Lipid peroxide levels showed a 51.85% decline relative to CCl_4_-treated rats. Super oxide dismutase (SOD), total antioxidant status (TAS), and catalase (CAT) revealed a marked increase by 132.48%, 187.18%, and 114.94% relative to the CCl_4_ group. In a tamoxifen-induced hepatotoxicity model, BIM showed a considerable alleviation in liver stress markers manifested by a 46.06% and 40% decline in ALT (Alanine Transaminase) and AST (Aspartate Transaminase) respectively. Thiobarbituric acid reactive substances (TBARS) were reduced by 28.57% and the tumor necrosis factor alpha (TNF-α) level by 50%. A virtual screening of major secondary metabolites of BIM to TNF-alpha employing the C-docker protocol showed that gmelinoside H caused the most potent TNF- α inhibition as indicated from their high fitting scores. Thus, BIM exhibited a potent hepatoprotective activity owing to its richness in antioxidant metabolites.

## 1. Introduction

The liver is one of the most crucial organs within the human body, being important for functions such as protein synthesis, as well as lipid and drug metabolism [1,2]. Hepatic diseases such as hepatitis, hepatocellular carcinoma, and cirrhosis from alcohol consumption have global relevance. They have been recognized as one of the serious causes of morbidity and mortality in humans [3]. The liver can also suffer from xenobiotics and hepatotoxins, in addition to viral infection and medical drugs. The majority of these factors eventually result in a dramatic burst of free radicals causing oxidative stress that impairs the metabolic functions of the liver [4].

Many of the synthetically manufactured drugs that are widely employed in the alleviation of hepatic disorders can exhibit adverse effects. Hence, the search for safer alternatives with fewer side effects is urgent. Medicinal plants that produce a wide diversity of secondary metabolites (PSM), could be useful in this context. In particular plant drugs that are rich in phenolic compounds, often exhibit antioxidant and liver protective properties [5,6]

The family Scrophulariaceae includes nearly around 87 genera with about 4800 species. Several taxa are used in traditional medicine and phytotherapy because of their antimicrobial, antioxidant, anti-inflammatory, hepatoprotective, and antihyperglycaemic properties. The family is also known for its richness in secondary metabolites particularly alkaloids, phenylpropanoids, iridoid glucosides, and terpenoids [7].

*Buddleia* (*Buddleja*) has recently been placed in Scrophulariaceae. It includes nearly 100 species native to Asia, Africa, as well as North and South America and several are cultivated worldwide, particularly in New Zealand and in central Europe [8,9]. Traditionally, the flowers of certain *Buddleia* species were eaten and cooked together with meat [10]. In addition, many medicinal properties, such as antimicrobial, antiviral, anti-inflammatory, hepatoprotective, antioxidant, antipyretic, analgesic, as well as immunosuppressive, antihypertensive, and antidiabetic activities have recently been assigned to members of the genus *Buddleia* [11,12,13,14,15,16,17,18,19]. Furthermore, various classes of bioactive secondary metabolites, such as flavonoids, phenylpropanoids, and iridoid glucosides have been isolated from *Buddleia* [20].

*Buddleia indica* Lam. (synonym *Nicodemia diversifolia* Ten.) is an evergreen shrub of African, specifically Madagascan origin. It is grown as an ornamental plant in many countries. Due to its oak-like leaf shape this taxon is known as Indoor Oak, however information on its biological activities or chemical constituents was not found in the literature [10].

In our project, we aimed to investigate the antioxidant and hepatoprotective activity of a methanol extract of *Buddleia indica* leaves (BIM) by both in vitro and in vivo studies. A phytochemical profiling of BIM using a LC/MS (Liquid Chromatography coupled with Mass Spectrometry) could identify the major secondary metabolites in the total methanol extract. Antioxidant activity was studied in vitro using the diphenyl picryl hydrazyl radicle scavenging capacity assay (DPPH•) and the hepatocarcinoma cell line HepG2 cells. For in vivo studies, we employed two experimental rat models: Hepatotoxicity was induced in rats by the application of chloroform (CCl_4_) and tamoxifen. Furthermore, molecular modeling of the major *Buddleia indica* PSM (plant secondary metabolites) was carried out with the tumor necrosis factor alpha (TNF-alpha) as a target.

## 2. Materials and Methods

### 2.1. Plant Material

*Buddleia indica* Lam. was obtained from El-Orman Botanical Garden, Giza, Egypt in 2016. The species was kindly identified and authenticated by Theresa Labib, Consultant of Plant Taxonomy at the Ministry of Agriculture and Director of the Orman Botanical Garden in Giza, Egypt. The voucher specimen of the obtained plant material (voucher number PHG-P-BI-163) is archived at the Pharmacognosy Department, Faculty of Pharmacy, Ain Shams University, Egypt.

### 2.2. Preparation of B. Indica Leaf Extract 

A total of 100 g of the air-dried leaves of *B. indica* were ground into coarse powder and then percolated with distilled methanol (1 L × 3) until exhaustion with subsequent filtration. The obtained filtrate was carefully evaporated under reduced pressure at 45 °C to complete dryness followed by lyophilization to yield 22.5 g of the dried total methanol extract (BIM).

### 2.3. LC-ESI-MS Profiling 

#### 2.3.1. HPLC Analysis

HPLC determination was carried out using 1 mg/mL of BIM, which was applied on Agilent 1100 HPLC Series (Agilent Technologies, Waldbron, Germany) using a Knauer column (250 × 4 mm ID) that was pre-packed with Eurospher 100-5. C18. The elution was done in a gradient manner starting with 10:90 of acetonitrile: H_2_O with 0.1% formic acid to 100% acetonitrile. The flow rate was 1 mL/min and one run took 35 min [21].

#### 2.3.2. Mass Spectrometry

Mass spectrometry was accomplished using a Finnigan LCQ-DECA mass spectrometer (ThermoFinnigan, San Jose, CA, USA), which was attached to a PDA detector. The BIM was dissolved in a mixture of H_2_O/MeOH followed by its direct injection into the HPLC/ESI-MS system. Negative ESI ionization ion mode was applied by adopting the following conditions: N_2_ was used as a drying and nebulizing gas; however the capillary temperature was kept at 250 °C. The spray voltage was 4.48 kV, the tube lens voltage was 10.00 V, and the capillary voltage was 39.6 V, however, a full scan mode was adjusted in mass range of *m*/*z* 100–2000 [21].

### 2.4. Biological Investigations 

#### 2.4.1. In Vitro Cytotoxicity 

##### Cell Cultures

Human lung cancer (A-549), human hepatic cancer (HepG2), and human prostate cancer (PC3) were obtained from VACSERA, Giza, Egypt. They were maintained in the tissue culture unit at the Pharmacology Department, Faculty of Pharmacy, Ain Shams University, Cairo, Egypt. Moreover, all the required media and the reagents needed for the cell culture reagents were supplied by Lonza (Basel, Switzerland). DMEM complete media were used to maintain the cell lines. The media contained 0.45% glucose, 10% of heat-inactivated bovine fetal serum albumin (FBS), 100 U/mL of penicillin, and 100 U/mL of streptomycin. Cells were cultured in cell culture dishes of 10 cm (Cellstar) at 37 °C in a humidified atmosphere of 5% CO_2_. Cells were grown as monolayer culture. All experiments were done using cells in the logarithmic growth phase.

##### Cytotoxicity and Cell Viability Assay

Cytotoxicity of BIM was assessed in HepG2, A-549, and PC3 cells utilizing the Sulforhodamine B cytotoxicity assay (SRB) exactly as mentioned before [22]. Briefly, cells growing in the exponential phase were collected with 0.25% of a mixture of trypsin and EDTA and placed in 96-well plates with 1000–2000 cells/well in RPMI-1640 supplied medium. After 24 h, cells were supplied with different concentrations of BIM for 72 h. Then 10% trichloroacetic acid was added for fixation and cells were maintained for 1 h and kept at 4 °C. Consequently, the staining of wells took place with 0.4% SRB in 1% acetic acid for 10 min at room temperature. Then they were carefully air-dried for 24 h followed by dissolution of the dye with Tris-HCl for 5 min on a shaker with 1600 rpm. For each well, the optical density (OD) was consequently determined spectrophotometrically at λ = 545 nm using an ELISA microplate reader (ChroMate-4300, Palm City, FL, USA). The standard anticancer drug, doxorubicin, was employed as a positive control. The IC_50_ was calculated as previously reported [22].

#### 2.4.2. Antioxidant and Hepatoprotective Activity

##### Chemical Reagents and Kits

2,2-Diphenyl-1-picrylhydrazyl (DPPH), silymarin, and CCl_4_ came from Sigma-Aldrich chemicals (St. Louis, MO, USA). Tamoxifen citrate (TAM) was bought from the Medical Union Pharmaceuticals Company (MUP), Cairo, Egypt. Aspartate transaminase (AST), alanine transaminase (ALT), reduced glutathione (GSH), superoxide dismutase (SOD) activity, and total antioxidant capacity (TAC) were determined by kits obtained from Biodiagnostics (Cairo, Egypt) and from Siemens Healthcare Diagnostics (Deerfield, IL, USA). The thiobarbituric acid for lipid peroxidation (LPO) determination was obtained from Fluka (Buchs, Switzerland) and the Quantikine^®^ rat TNF-α/TNFSF1A immunoassay kit to measure TNF-α was obtained from Bio-Techne Ltd. (Abingdon, Oxford, UK). 

##### In Vitro Antioxidant and Hepatoprotective Activity

Diphenyl Picryl Hydrazyl Radicle Scavenging Capacity Assay (DPPH•)

The DPPH assay was carried out in accordance with the published protocols [23] in which 50 μL of BIM at concentrations of 0.2, 0.4, 0.6, and 1.0 mg/mL was incubated separately with 5 mL of the methanol solution of 0.004% DPPH. After 30 min of incubation at room temperature, absorbance was measured directly using a Shimadzu UV-1601 spectrophotometer (Kyoto, Japan) at 517 nm. The positive control ascorbic acid showed a IC_50_ = 1.57 μg/mL. The percentage of inhibition of DPPH• was calculated using the following equation:

Free radical scavenging activity = [Ac − As/Ac] × 100

Ac: absorbance of control; As: absorbance of sample.

##### Antioxidant Activity in HepG2 Cells

BIM at concentrations of 0.01, 0.1, and 1 mg/mL was tested in vitro and concomitantly compared to silymarin. We used monolayer cultures of the liver cancer cell line HepG2 that were previously treated with the tested samples for 1 h or the positive control silymarin. Then 0.05% dimethyl sulfoxide containing 40 mM CCl_4_ was subsequently added to the cells and incubated for 2 h more. Then both the cell lysate and the supernatant of the medium were collected and kept until analysis at −20 °C. The untreated control consisted of cells that were maintained in phosphate-buffered saline only. ALT and AST levels were evaluated by spectrophotometry (546 nm) in the supernatant using commercially available kits obtained from Biodiagnostics (Cairo, Egypt) [24]. Superoxide dismutase (SOD), reduced glutathione (GSH), and total antioxidant capacity (TAC) were determined spectrophotometrically in cell lysates using the methods previously described by Ellman [25], Nishikimi et al. [26], and Koracevic et al. [27] respectively. In the in vitro study, we selected doses in an exponential increase to evaluate the effect on a crystal clear base. We do understand that the 1 mg dose was very high but it was selected to show the maximum effect that could be obtained. However, our main targets were 0.01 and 0.1 mg doses. These doses were selected based on several published data in the same field of assessing antioxidant activities [28,29]. It is notworthy to highlight that the in vitro studies act only as preliminary tools for further in vivo investigations using animal models and should be consolidated by clinical trials to declare its significance in treatment of human disorders. 

##### In Vivo Antioxidant and Hepatoprotective Assessment

##### Animals and Animal Treatment

Male Wister rats (170–260 g) and 120–140 g adult female Sprague—Dawley rats were supplied from the animal facility of King Fahd Medical Research Center, King Abdulaziz University (Jeddah, Saudi Arabia) and were used to carry out the in vivo experiments using carbon tetrachloride and tamoxifen as hepatotoxic agents, respectively. The protocol for the experiments was accepted by the local ethical committee, Taibah University (Jeddah, Saudi Arabia) (TUCDREC/20160131). The animals were maintained at standard temperature (24 ± 5 °C), 55 ± 5% relative humidity and a 12 h light, 12 h dark cycle. Additionally, rats had free access to standard food (Purina Chow) and water. Moreover, they were treated in a humane manner that adhered with the animal care guideline of WHO (World Health Organization) [30].

##### LD_50_ Experiment

LD_50_ experiment was carried out with adult male Wister rats, weighing 170–260 g in order to estimate the acute toxicity of the BIM extracts at different concentrations. Animals were observed for 20 consecutive days and accordingly, safety doses were calculated. Doses that were considered as safe were doses below 100 mg/kg b.wt.

##### In Vivo Assessment of the Hepatoprotective Activity in CCl_4_ Induced Hepatotoxicity Model

Male Wister rats were classified into 4 groups with each group comprised of 10 rats. Animals of the first group were given normal saline (p.o.) (untreated control). Rats in the second group were treated (p.o.) with equal volumes of CCl_4_ and paraffin oil (1 mL/kg b.wt. for 20 days) [31]. CCl_4_-treated rats in group 3 obtained a daily oral dose of 50 mg/kg b.wt. of silymarin for 20 days. In group 4, the CCl_4_-treated animals obtained an oral dose of 50 mg BIM/kg b.wt. for 20 days. The liver enzymes for group 2 were evaluated in sera after 72 h of CCl_4_ application in order to confirm the onset of liver toxicity. At the end of the experiment, the rats were anesthetized by ether and blood was taken from the optical vein using a capillary tube. The blood was collected in polypropylene tubes, followed by centrifugation at 4000 rpm to separate sera. All samples were frozen at −20 °C until assayed.

##### Hepatoprotective Activity of BIM in the Tamoxifen Induced Hepatotoxicity Model

The experimental animals (adult female Sprague—Dawley) were distributed randomly to 4 groups, with 8 rats in each group. The first group (control) was treated (i.p.) with normal saline for 7 days. Group 2 was injected (i.p.) daily with 20 mg of tamoxifen /kg b.wt. (TAM) for 7 days. In groups 3 and 4, the TAM-animals were treated (i.p.) with silymarin and BIM for 7 days in doses similar to those in the second group. After 24 h of the last treatment, the rats were anesthetized with ether and blood was then collected and processed as in the chloroform experiments. ALT and AST levels were assessed in sera. For further investigations, rats were killed by cervical dislocation. The liver was removed and washed in ice-cold isotonic saline. Liver tissues were in ice-cold 0.1 M of potassium phosphate buffer (pH 7.5), and stored at −70 °C for subsequent analysis [22].

##### Evaluation of the Biochemical Parameters

##### Estimation of ALT and AST Activities

Spectrophotometric diagnostic kits (Siemens Healthcare Diagnostics, Deerfield, IL, USA) were employed to measure the AST and ALT levels using an autoanalyzer (Dimension^®^ Xpand^®^, Siemens Healthcare Diagnostics, Deerfield, IL, USA).

Estimation of Serum Lipid Peroxides (LPOs)

LPOs in the serum were determined in the form of thiobarbituric acid reactive substances (TBARS) (nmol/mL). In brief, 1 mL of 1% H_3_PO_4_ was added to 1 mL of 6% TBARS in 0.25M HCl that were shaken with 0.5 mL of serum and maintained in boiling water for 50 min. Immediately after cooling, *n*-butanol (4 mL) was subsequently added. After centrifugation at 6000 rpm/min for 20 min, the *n*-butanol layer was separated and spectroscopy was carried out at 535 and 520 nm [32,33].

##### Estimation of Serum TAS

A total of 20 μL of the serum and 1 mL of chromogen were shaken together in a cuvette. The chromogen consisted of 610 mmol/L 2,2’-azino-di-(ethylbenzthiazoline sulphonate) together with 6.1 mmol/L of metmyoglobin. The initial absorbance (A_1_) was recorded. A total of 200 μL of the substrate 250 mmol/L H_2_O_2_ was added and after 3 min the absorbance (A_2_) was determined and ∆A was calculated [34]. TAS (mmol/L) = factor × (∆A blank − ∆A sample). Factor = concentration of standard/(∆A blank − ∆A standard).

##### Estimation of Serum Catalase

The activity of catalase was measured using 2 mL of the reaction mixture that consisted of 1.95 mL of 10 mM hydrogen peroxide (H_2_O_2_) in 60 mM of phosphate buffer (pH 7). Initiation of the reaction was achieved via the addition of 0.1 mL of serum and then absorbance was measured at 240 nm after 3 min. One CAT unit was identified as the quantity of H_2_O_2_ changed to H_2_O and ½ O_2_ in 1 min applying standard conditions and then the specific activity was recorded as U/mL [35].

##### Estimation of Serum SOD

The superoxide radicals generated in a reaction between 50 mM of xanthine oxidase (1000 U) and 50 mM of xanthine, 0.1 mM of EDTA, and 50 mM of NBT produced a red formazan dye which could be determined in a spectrophotometer at 250 nm. To measure the SOD in the sample, which will compete for superoxide radicals, 0.1 mL of serum was added. The activity of SOD was measured in U/mL [36].

##### Estimation of Tumor Necrosis Factor-Alpha (TNF-α)

TNF-α was determined in the liver homogenate utilizing the Quantikine^®^ rat TNF-α/TNFSF1A immunoassay kit (Bio-Techne Ltd., Abingdon, Oxford, UK). The liver homogenate was added to a microplate that was coated with a monoclonal antibody, which specifically binds rat TNF-α. A polyclonal antibody that specifically binds rat TNF-α (conjugated to horseradish peroxidase) was added after washing. After removing the excess of antibodies, tetramethylbenzidine was added and incubated for a period of 30 min. The color was determined spectrophotometrically at 450 nm.

All measurements were done spectrophotometrically using a Shimadzu UV-1601 spectrophotometer (Kyoto, Japan).

##### Statistical Analysis

Results were recorded as mean ± SEM (standard error of the mean). One-way analysis of variance (ANOVA) was adopted for statistical analysis. Post hoc Tukey’s test was done to further compare the groups in condition at *p* < 0.05. Statistical analysis and sketching of graphs were accomplished using GraphPad Prism version 5 software (GraphPad Software, Inc. La Jolla, CA, USA).

### 2.5. Molecular Modelling Studies

A virtual screening of the major PSM from BIM on the tumor necrosis factor alpha (TNF-alpha) was done employing Discovery Studio 2.5 (Accelrys Inc., San Diego, CA, USA) and the C-docker protocol implementing both the rule- and pH-based ionization method. The structure of the TNF-alpha (PDB ID 2AZ5; 2.1 Å) was downloaded from a protein data bank (www.pdb.org) [37]. PSM were docked to the active site and the binding energies for docking were recorded as previously mentioned [38,39,40].

## 3. Results

In this communication, we performed LC-ESI-MS profiling of *Buddleia indica* leaves (BIM) to identify its metabolites. In addition an in-depth biological study was carried out both in vitro and in vivo in order to find solid evidence of the hepatoprotective activity of the plant extract. It started with the in vitro study by antioxidant determination using DDPH scavenging capacity assay as well as in vitro HepG2 cell model to in vitro assess the potential hepatoprotective activity of the plant extract as a preliminary screening to get an overview about the activity. Then, different models with different induction methods were chosen for assessing such activity that are carbon tetrachloride induced hepatotoxicity and tamoxifen induced hepatic damage in rat models.

### 3.1. LC-ESI-MS Analysis of BIM

**Figure 1:**
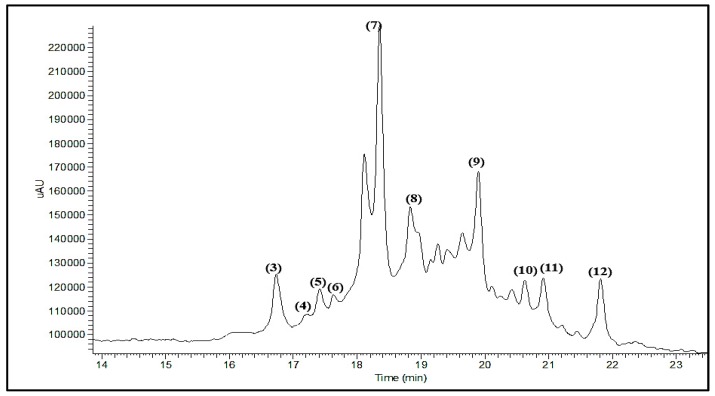
LC-ESI-MS (Liquid Chromatography coupled with Electrospray Ionization Mass Spectrometry) profiling of the total methanol extract of *Buddleia indica* leaves, the numbers represent the peaks number that correspond to the compounds number.

LC-ESI-MS profiling of *Buddleia indica* leaves (BIM) (Figure 1) revealed 12 main peaks, which were tentatively identified by comparing their MS data (in the negative ionization mode) and their UV/Vis spectra with previously recorded PSM in the literature (Table 1).

**Figure 2:**
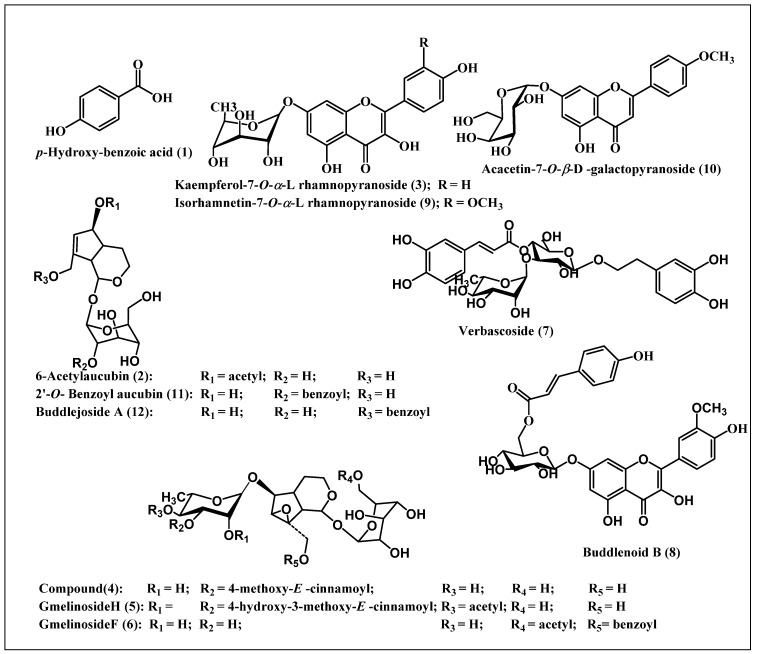
A scheme representing the compounds identified from the total methanol extract of *Buddleia indica* leaves.

The identified compounds belong mainly to the classes of iridoids, phenylpropanoids, and flavonoids (Table 1) and comprise of *p*-hydroxy-benzoic acid **(1)** [41], 6-acetylaucubin **(2)** [42], kaempferol-7-o-*α*-l rhamnopyranoside **(3)** [43], catalpol 6-o-[4-methoxy-E-cinnamoyl-(3)-α -l-rhamnopyranoside **(4)** [44], gmelinoside H **(5)** [45], gmelinoside F **(6)** [45], verbascoside **(7)** [46], buddlenoid B **(8)** [47], isorhamnetin 7-o-*α*-l rhamnopyranoside **(9)** [48], acacetin7-galactoside **(10)** [49], 2′-o-benzoyl aucubin **(11)** [50], and buddlejoside A **(12)** [51]. Structures of the identified compounds are provided in Figure 2. Compounds **2**, **4**, **5**, **6**, **11**, and **12** are iridoid glycosides however compounds **3**, **8**, **9**, and **10** belong to flavonoids whereas compound **7** is a phenylpropanoid.

### 3.2. Pharmacological Investigations

#### 3.2.1. Evaluation of BIM Cytotoxic Activity

BIM exhibited a very weak or even no cytotoxic activity against all of the examined tumor cell lines, namely HepG2, A-549, and PC3 (Table 2) that revealed its apparent safety.

#### 3.2.2. In Vitro Antioxidant and Hepatoprotective Activity

The DPPH assay revealed that BIM exerts mild antioxidant activity with an IC_50_ value of 0.43 mg/mL. In HepG2 cells, which were treated with CCl_4_ to induce membrane damage, an application of 0.01, 0.1, and 1 mg/mL counteracted the toxic effects in a concentration dependent manner. This was reflected by the normalization of AST and ALT levels that reached 47.90 and 66.52 U/mL at 1 mg/mL of BIM, respectively. In addition, 1 mg/mL of BIM also caused a restoration of GSH level as well as SOD activity and TAC revealing 16.82 mg/dL, 353.6 U/mL, and 1.51 nmol/mL, respectively. As shown in Table 3, BIM exhibited similar protective activities as silymarin, the known hepatoprotective agent from *Silybum marianum*. 

#### 3.2.3. In Vivo Antioxidant and Hepatoprotective Activity in CCl_4_ Rat Model

**Figure 3:**
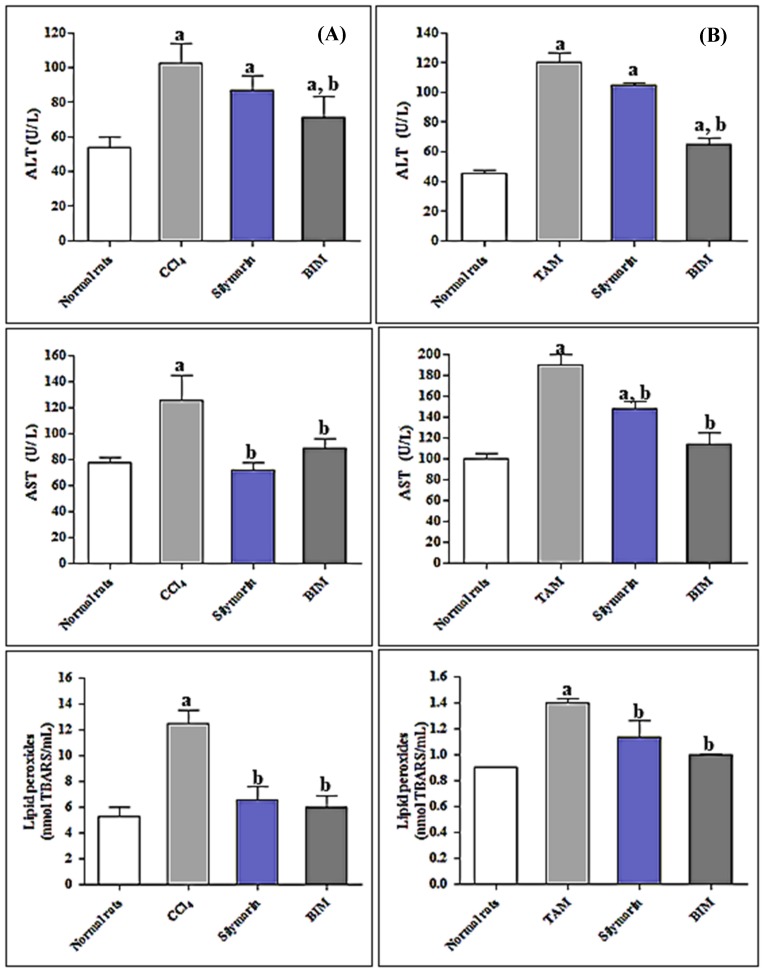
Influence of treatment with BIM extract on ALT, AST, and lipid peroxides level in (**A**) CCl_4_ treated rats and (**B**) tamoxifen citrate (TAM) treated rats. Results are expressed as means ± S.E.M. (*n* = 10). ALT and AST: Measured using spectrophotometric diagnostic kits. Lipid peroxidases: Measured spectrophotometrically at 535 nm. ^a^ Significantly different from normal control (*p* < 0.01); ^b^ Significantly different from CCl_4_/TAM control (*p* < 0.01).

The oral administration of 1 mL of CCl_4_ /kg body weight (BW) to rats resulted in a substantial rise in the serum levels of AST and ALT by 61.09% and 91.59%, with respect to the control group (Figure 3). Concomitantly, a marked reduction in serum levels of oxidative stress markers was observed with TAS, SOD, and CAT decreasing by 63.75%, 59.53%, and 71.53%, respectively compared to the control group. Additionally, the generation of lipid peroxides was greatly enhanced by 135.54% as compared to control rats.

However, administration of silymarin to CCl_4_-treated group resulted in a pronounced reduction in the serum levels of ALT and AST by 15.03% and 42.73%, respectively with concomitant elevation in SOD, CAT, as well as TAS by 102.82%, 125.64%, and 103.44%, respectively. Furthermore, treatment with silymarin resulted in a significant decline in lipid peroxide level in the CCl_4_-treated group. Regarding treatment with BIM, its oral administration at a dose of 50 mg/kg of BW resulted in a pronounced reduction of liver stress markers where ALT and AST serum levels showed a 30.63% and 29.44% reduction, respectively as compared to CCl_4_-treated rats. In addition, the oxidative stress markers were significantly ameliorated after the oral administration of BIM. Lipid peroxide levels showed a 51.85% decline with respect to CCl_4_-rats (Figure 3A).

SOD, CAT, and TAS showed marked amelioration after oral BIM administration (132.48%, 187.18%, and 114.94%, respectively relative to CCl_4_-rat) which exceeded the values for silymarin and approached those of the untreated control group (Table 4).

#### 3.2.4. In Vivo Antioxidant and Hepatoprotective Activity of BIM towards Tamoxifen Induced Hepatotoxicity

At a dose of 20 mg/kg of BW tamoxifen (TAM) increased the liver stress markers, namely ALT and AST, by 164.79% and 89.99% as compared to the untreated control group. TAM caused a notable elevation in TBARs (by 55.55%) and concomitantly in TNF-α level by 337.5% as compared to the untreated controls. BIM reduced tamoxifen toxicity as the liver stress markers ALT and AST declined by 46.06% and 40%, compared to the 12.93% and 22.45% decline by silymarin. In addition, BIM considerably ameliorates the oxidative stress caused by TAM as evidenced by reducing TBARs by 28.57% and TNF-α level by 50% indicating its higher potency than silymarin that causes a 19.29% and 20% reduction in the TBARs and TNF-α level, respectively with respect to TAM rats (Figure 3B and Table 5).

### 3.3. In Silico Molecular Modeling Studies

**Figure 4:**
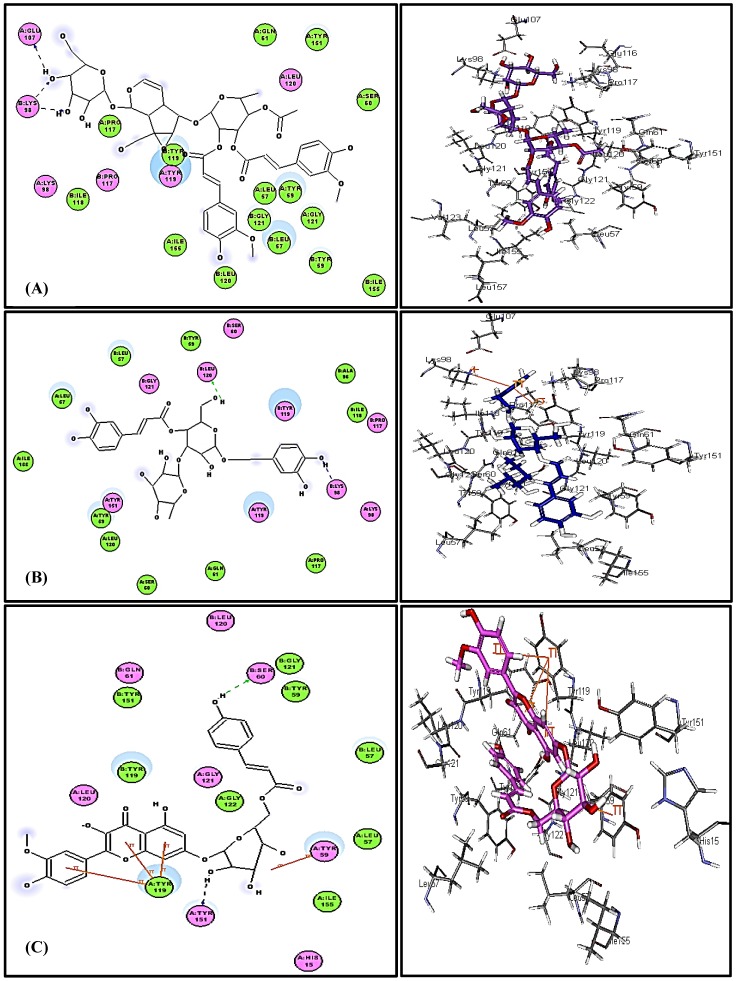
2D and 3D binding mode of (**A**) GmelinosideH, (**B**) Verbascoside, and (**C**) Buddlenoid B in the active site of TNF-α.

**Figure 5:**
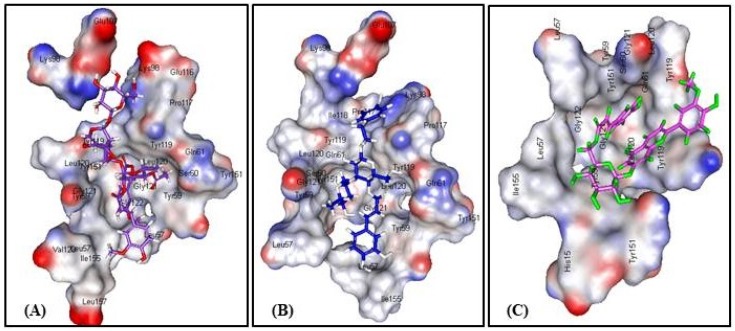
Alignment of (**A**) Gmelinoside H, (**B**) Verbascoside, and (**C**) Buddlenoid B in the active pocket of TNF-α.

Virtual screening showed that the iridoid glycoside gmelinoside H **(5)** revealed the strongest affinity to TNF-α as indicated from its discriminant fitting score (59.66 and −62.58 Kcal/mol binding energies) in both the pH and rule based ionization modes that undoubtedly reflect its pronounced stability in the active pockets which are comparable to the original co-crystallized ligand (L) (Table 6). In addition, the predominant compounds in the extract represented by the phenylpropanoid verbascoside **(7)** and the flavonoid glycoside, buddlenoid B **(8)**, also exhibited a substantial binding within the active sites exceeding that of the original ligand revealing free binding energies of −53.20 and −48.73 Kcal/mol in the pH based ionization mode and −53.64 and −49.37 Kcal/mol in the rule based ionization mode, respectively. The 2D and 3D binding modes of the compounds that showed the highest fitting within the active sites were illustrated in Figure 4. The alignment of the potent compounds in the pocket of the enzyme active site is documented in Figure 5.

## 4. Discussion

LC-ESI-MS profiling indicate the presence of 12 main compounds belonging mainly to iridoids, phenylpropanoids, and flavonoids (Table 1, Figure 2). Verbascoside and buddlenoid B represent the main predominating compounds, with the former being previously identified in many *Buddleia* species as *B. cordata*, *B. globose*, and *B. davidii* [47,52,53] whereas the latter was isolated from *B*. *coriacea* [46]. Additionally, other identified compounds include buddlejoside A, which was previously identified in *B. crispa* and *B. japonica* [51,54].

Meanwhile the flavonoids acacetin-7-o-β-d-galactoside, isorhamnetin-7-o-α-L rhamnopyranoside, and kaempferol-7-o-α-L rhamnopyranoside are considered as various glycoside derivatives of the aglycones already isolated from multiple *Buddleia* species [46,55]. The iridoids 6-acetylaucubin, 2′-o-benzoyl aucubin, and catalpol 6-o-[4-methoxy-E-cinnamoyl-(3)-α -l-rhmnopyranoside are considered derivatives of aucubin and catalpol, which are widely distributed in the genus [56,57]. However, the two acylated iridoids, gmelinoside H and gmelinoside F exist as minor components and are new PSM for the genus.

Herein, we used CCl_4_ and tamoxifen to induce hepatotoxicity in rats, which exert their toxicity by ROS generation. CCl_4_ is bio-transformed by the liver cytochrome P450s into trichloromethyl free radicals that ultimately cause lipid peroxidation and tissue destruction [22]. Additionally, tamoxifen initiates the generation of ROS that causes oxidation of DNA base pairs with ultimate cell and protein damage [58]. Evidence for liver damage is a strong increase of serum ALT and AST and modulation of other oxidative stress markers (Table 3, Figure 3).

BIM showed a potent antioxidant and hepatoprotective activity that is more pronounced in rats than in vitro (Table 3, Figure 3). In vitro studies were done as a preliminary tool to screen the bioactivity of natural products that should be followed by in vivo as well as clinical trials. It is noteworthy to highlight that treating hepatocytes with a crude extract (i.e., BIM) is not physiologically relevant because hepatocytes (under in vivo conditions) will never be exposed to an extract in its entirety because the extract is not absorbed intact but rather select compounds are absorbed at the intestine (they are also likely to undergo extensive xenobiotic metabolism). In vitro studies are helpful to initiate in vivo studies acting as a preliminary tool to predict the activity and it also acts as a guide for the choice of in vivo protocol as the selection of carbon tetrachloride to induce hepatotoxicity in the current study. 

There are many limitations of the in vitro studies relative to the in vivo studies because the mechanism of action under in vitro conditions (as tested) is likely to be quite different from that occurring in vivo. Furthermore, a BIM extract contains many antioxidant compounds as manifested from its LC/MS profile represented by flavonoids, irridoids, and phenylpropanoids that possess free hydroxyl groups. These antioxidant compounds are more likely to stimulate Nrf2 (Nuclear factor erythroid 2-related factor 2) which in turn control the expression of various antioxidant proteins that is crucial for protecting versus oxidative damage caused by injury as well as inflammation. They may behave in a different manner under in vitro conditions comparable to the in vivo conditions [59]. 

Meanwhile, the potential in vivo activity of BIM may be attributed to the activation of certain metabolites in rats [30]. For example, iridoid glucosides (which are abundant in BIM) are regarded as prodrugs, which become activated after glucosidic cleavage (van Wyk and Wink, 2015, 2017). The aglycone is unstable: The lactol rings open and two reactive aldehyde groups are formed, which can covalently bind to amino groups in proteins (van Wyk and Wink, 2015, 2017). BIM caused significant amelioration in ALT and AST in both CCl_4_ and TAM rats exceeding that of the known liver protectant silymarin. Levels of lipid peroxides, SOD, CAT, and TAS are considerably alleviated upon BIM treatment accompanied by a concomitant normalization of TNF-α, one of the known inflammation markers-α (Table 4, Figure 3). 

BIM contains PSM, especially phenylpropanoids, iridoids, and flavonoids, with a known history of antioxidant and anti-inflammatory activity, relevant to oxidative liver damage. Phenolic PSM effectively antagonize the dangerous effects of ROS via the inhibition of the superoxide anion generating enzymes as lipoxygenase, glutathione S-transferase, protein kinase C, NADH oxidase, mitochondrial succinoxidase, and xanthine oxidase. Moreover, they can act as transition metal chelators through their adjacent phenolic-OH groups which function as free radical scavengers and thus inhibit free radical chain reactions [60,61]. BIM showed considerable anti-inflammatory activity evidenced from the reduction of TNF-α, one of the relevant inflammatory markers. Flavonoids and iridoid glucosides have been regarded as anti-inflammatory agents in many medicinal plants [62,63]. Our molecular modeling study supports this assumption: The iridoid glucoside gmelinoside H showed the highest inhibition owing to the formation of three firm hydrogen bonds with Glu 107 and Lys 98 amino acid residues at the active site. Regarding verbascoside and buddlenoid B, they also showed considerable inhibition via the formation of two firm hydrogen bonds with Leu 120 and Lys 98 for the formatter, while buddlenoid B formed two hydrogen bonds with Ser 60 and Tyr151 and three π- bonds with Tyr 119.

Meanwhile standardization of BIM extract using HPLC experiments with markers, LC/MS techniques, and more recently coupled with chemometrics is mandatory and should be done between carrying different studies since considerable differences in BIM preparations could yield a very different phytochemical profile and consequently different pharmacological findings.

However, we aim in our future work to isolate the major constituents of the BIM extract in their pure single forms and examine the biological activity of each pure single compound both in vivo and in vitro to further confirm the activity of the whole extract (BIM) that could be achieved through the synergistic activity of its compounds. 

## 5. Conclusions

Our in vitro and in vivo data indicate that the extracts from *B. indica* leaves could be regarded as a potent antioxidant and hepatoprotective agent. As the extract was rich in antioxidant phenolic compounds, its bioactivity against hepatotoxins is plausible. The extract could counteract liver inflammation and reduce TNF-α, which was further supported by molecular modeling studies. Iridoid glucosides are known anti-inflammatory compounds in several medicinal plants. More studies are required to ascertain the obtained results and to see if the BIM might be applied in phytotherapy using different models as NAFLD (nonalcoholic fatty liver disease) and NASH (nonalcoholic steatohepatitis). Additionally, tracing the metabolism of the identified PSMs both in vitro and in vivo to understand the roles of the metabolites in contributing to the health benefits reported in the study is also recommended.

## Figures and Tables

**Table 1:** Identification of the major secondary metabolites in the total methanol extract of *Buddleia indica* leaves (BIM) applying the LC-ESI-MS (Liquid Chromatography coupled with Electrospray Ionization Mass Spectrometry) technique.

No.	*R*_t_ (min)	UV (λmax) (nm)	(M-H)^−^ *m*/*z*	Compounds	References
1	1.36	248	136.95	*p*-Hydroxy-benzoic acid	[41]
2	1.62	252	387.13	6-Acetylaucubin	[42]
3	16.37	242,330,	430.79	Kaempferol-7-o-α-l rhamnopyranoside	[43]
4	17.15	244,292,330	667.55	Catalpol-6-o-[4-methoxy-e-cinnamoyl-(3)-α -l-rhamnopyranoside	[44]
5	17.32	244,306,330	901.62	Gmelinoside H	[45]
6	17.60	242,316	669.43	Gmelinoside F	[45]
7	18.47	242,330	623.41	Verbascoside	[46]
8	18.96	242,330	623.31	Buddlenoid B	[47]
9	19.93	244,330	461.29	Isorhamnetin-7-o-α-L rhamnopyranoside	[48]
10	20.47	244,330	445.15	Acacetin-7-o-β-d galactoside	[49]
11	20.95	244,300,328	449.06	2′-o-Benzoyl aucubin	[50]
12	21.77	238,303	465.50	Buddlejoside A	[51]

**Table 2:** IC_50_ values (µg/mL) for the cytotoxic effects of BIM on the growth of different cancer cells.

Cell Line	BIM (µg/mL)	Doxorubicin (µg/mL)
A549	>1000	0.48 ± 0.031
PC3	207.3 ± 19.00	0.49 ± 0.039
HepG2	657.7 ± 56.01	0.22 ± 0.020

Data are presented as means ± S.D. (measured in triplicates; *n* = 3). Using SRB (Sulforhodamine B cytotoxicity assay) and measured spectrophotometrically at 564 nm.

**Table 3:** Effect of BIM on hepatic toxicity markers (aspartate transaminase: AST and alanine transaminase: ALT), and oxidative stress markers (glutathione: GSH, superoxide dismutase: SOD, and total antioxidant status: TAC) levels in HepG2 cells exposed to CCl_4_.

Groups	AST ^a^ (U/mL)	ALT ^a^ (U/mL)	GSH ^b^ (mg/dL)	SOD ^c^ (U/mL)	TAC ^d^ (nmol/mL)
Control	26.77 ± 1.68 *	59.20 ± 0.95 *	16.81 ± 0.08 *	384.7 ± 13.50 *	0.82 ± 0.016 *
CCl_4_	57.23 ± 1.87	85.36 ± 1.69	12.65 ± 0.29	251.0 ± 4.80	0.13 ± 0.014
CCl_4_ + Silym (0.01 mg/mL)	45.25 ± 0.82 *	73.59 ± 1.06 *	14.99 ± 0.06 *	334.01 ± 6.41 *	0.88 ± 0.010 *
CCl_4_ + Silym (0.1 mg/mL)	41.57 ± 1.24 *	67.09 ± 1.88 *	16.59 ± 0.09 *	384.01 ± 18.92 *	0.99 ± 0.012 *
CCl_4_ + Silym (1 mg/mL)	35.04 ± 1.54 *	60.05 ± 1.75 *	18.43 ± 0.17 *	412.3 ± 3.11 *	1.28 ± 0.033 *
CCl_4_ + BIM (0.01 mg/mL)	57.10 ± 1.41	77.57 ± 0.81 *	15.51 ± 0.71 *	294.6 ± 12.40	0.90 ± 0.013 *
CCl_4_ + BIM (0.1 mg/mL)	53.77 ± 2.36	73.02 ± 1.88 *	15.93 ± 0.42 *	326.8 ± 13.50 *	1.09 ± 0.022 *
CCl_4_ + BIM (1 mg/mL)	47.90 ± 0.42 *	66.52 ± 1.53 *	16.82± 0.55 *	353.6 ± 8.20 *	1.51 ± 0.028 *

Data are presented as means ± S.E.M. (measured in triplicates; *n* = 3), * significantly different from CCl_4_ at *p* < 0.05, **^a:^** Measured spectrophotometrically at 546 nm using spectrophotometric diagnostic kits, **^b:^** Measured colorimetrically at 412 nm, **^c:^** Measured colorimetrically at 420 nm, and **^d:^** Measured colorimetrically at 505 nm.

**Table 4:** Effect of BIM on TAS, SOD, and CAT levels in vivo the CCl_4_-induced hepatotoxicity model.

Groups	* TAS (mmol/L)	* SOD (U/mL)	* CAT (U/mL)
Normal Control	7.20 ± 0.63 ^b^	103.30 ± 1.7 ^b^	1.37 ± 0.21 ^b^
CCl_4_ treated group	2.61 ± 0.32 ^a^	41.81 ± 4.12 ^a^	0.39 ± 0.03 ^a^
CCl_4_-treated rats + Silymarin	5.31 ± 0.43 ^a,b^	84.8 ± 5.95 ^a,b^	0.88 ± 0.04 ^a,b^
CCl_4_-treated rats + BIM	5.61 ± 0.72 ^a,b^	97.2 ± 7.83 ^b^	1.12 ± 0.05 ^b^

Results are expressed as means ± S.E.M. (*n* = 10). * Measured spectrophotometrically. ^a^ Significantly different from normal control (*p* < 0.01). ^b^ Significantly different from CCl_4_ control (*p* < 0.01).

**Table 5:** Effect of BIM on tumor necrosis factor alpha (TNF-α) level in vivo tamoxifen (TAM) induced hepatotoxicity model.

Groups	TNF-α (pg/g Protein)
Normal Control	160 ± 11 ^b^
TAM treated group	700 ± 14 ^a^
TAM-treated rats + Silymarin	560 ±11 ^a,b^
TAM-treated rats + BIM	350 ± 10 ^a,b^

Results are expressed as means ±S.E.M. (*n* = 10). ^a^ Significantly different from normal control (*p* < 0.01). ^b^ Significantly different from TAM control (*p* < 0.01).

**Table 6:** Free binding energies (ΔG) of the identified PSM (plant secondary metabolites) in BIM to TNF-alpha active sites in kcal/mol adopting both pH and rule based ionization techniques.

Compound	pH-Based	Rule-Based
*p*-Hydroxy-benzoic acid **(1)**	−20.42	−19.61
6-Acetylaucubin **(2)**	FD	−39.19
Kaempferol-7-O-*α*-L rhamnopyranoside **(3)**	−40.38	−38.34
Catalpol-6-O-[4-methoxy-E -cinnamoyl-(3)-α -L-rhamnopyranoside **(4)**	−53.30	−51.88
GmelinosideH **(5)**	−59.66	−62.58
GmelinosideF **(6)**	−48.12	−56.31
Verbascoside **(7)**	−53.20	−53.64
Buddlenoid B **(8)**	−48.73	−49.37
Isorhamnetin-7-O-*α*-L rhamnopyranoside **(9)**	−37.74	−40.56
Acacetin-7-galactoside **(10)**	FD	FD
2’-*O*-Benzoyl aucubin **(11)**	FD	−39.72
Buddlejoside A **(12)**	FD	−35.88
Ligand	−45.74	−44.97

FD: fail to dock.
